# The Effect of Strain Rate on Bone Fragility and Clinical Applications

**DOI:** 10.3390/bioengineering12121295

**Published:** 2025-11-24

**Authors:** Marijo Bekić, Petra Bagavac, Nenad Šešić, Marijana Kulić, Antun Bekić

**Affiliations:** 1 Dubrovnik County Hospital, 20000 Dubrovnik, Croatia; marijob@bolnica-du.hr (M.B.); nenad.sesic@yahoo.com (N.Š.); 2 Faculty of Electrical Engineering, Mechanical Engineering and Naval Architecture, University of Split, 21000 Split, Croatia; 3 Medical School Dubrovnik, 20000 Dubrovnik, Croatia; marijanakulic77@gmail.com; 4 School of Medicine, Catholic University of Croatia, 10000 Zagreb, Croatia; bekicantun@gmail.com

**Keywords:** bone fracture mechanism, dynamic tests, impact velocity, ductility index

## Abstract

The loading rate influences the fracture mechanics of bone. The effect of strain rate on bone tissue was studied. Two sets of dynamic tests were performed, where the impact energy E = 70 J was kept as a constant value. In the first set, the impact velocity of the striker was set to 5.15 m/s, and in the second set of dynamic tests, the impact velocity was set to 7.84 m/s. In each set, four pig femurs were tested, resulting in a total of eight femurs tested. In order to maintain the same impact energy, when testing at a lower velocity, a set of weights (+3 kg) was added to the impactor. The ductility index evaluates the propagation behaviour of brittle versus ductile fracture based on direct force measurements from a force/time diagram.

## 1. Introduction

Mechanical properties of bone are governed by a combination of its structural architecture and the intrinsic characteristics of the constituent tissue. Although the outcomes of biomechanical measurements are typically reported in standard SI units, analogous to engineering materials, cortical and cancellous bone cannot be meaningfully represented as two independent mechanical domains. Their behaviour reflects an integrated mechanical system whose geometry and composition are biologically optimized for efficient energy absorption. Consequently, assigning distinct elastic moduli to cortical and cancellous regions provides only an approximate description of the overall mechanical response. Similarly, while the term anisotropic is widely employed in biomechanical analyses, it inadequately describes the full complexity of bone’s hierarchical and biologically adapted behaviour.

Loading rate is a further determinant of fracture mechanics. At low loading rates, bone generally exhibits a more ductile fracture pattern, whereas higher loading rates promote brittle or comminuted failure due to the restricted time available for elastic deformation. In addition, microdamage accumulated during mechanical testing contributes to degradation of bone quality, thereby increasing fragility and the probability of failure.

Fractures are typically classified as brittle when the ductility index is below 30%, and as ductile when the ductility index exceeds 60%. In this context, the working hypothesis is that different impact velocities induce distinct bone fracture mechanisms. It is assumed that lower impact velocities will allow a longer interval for elastic deformation, resulting in a ductile fracture response. Conversely, higher impact velocities are expected to produce brittle fractures. The ductility index is employed to quantitatively assess the governing fracture mechanism under varying loading conditions.

## 2. Materials and Methods

**Figure 1:**
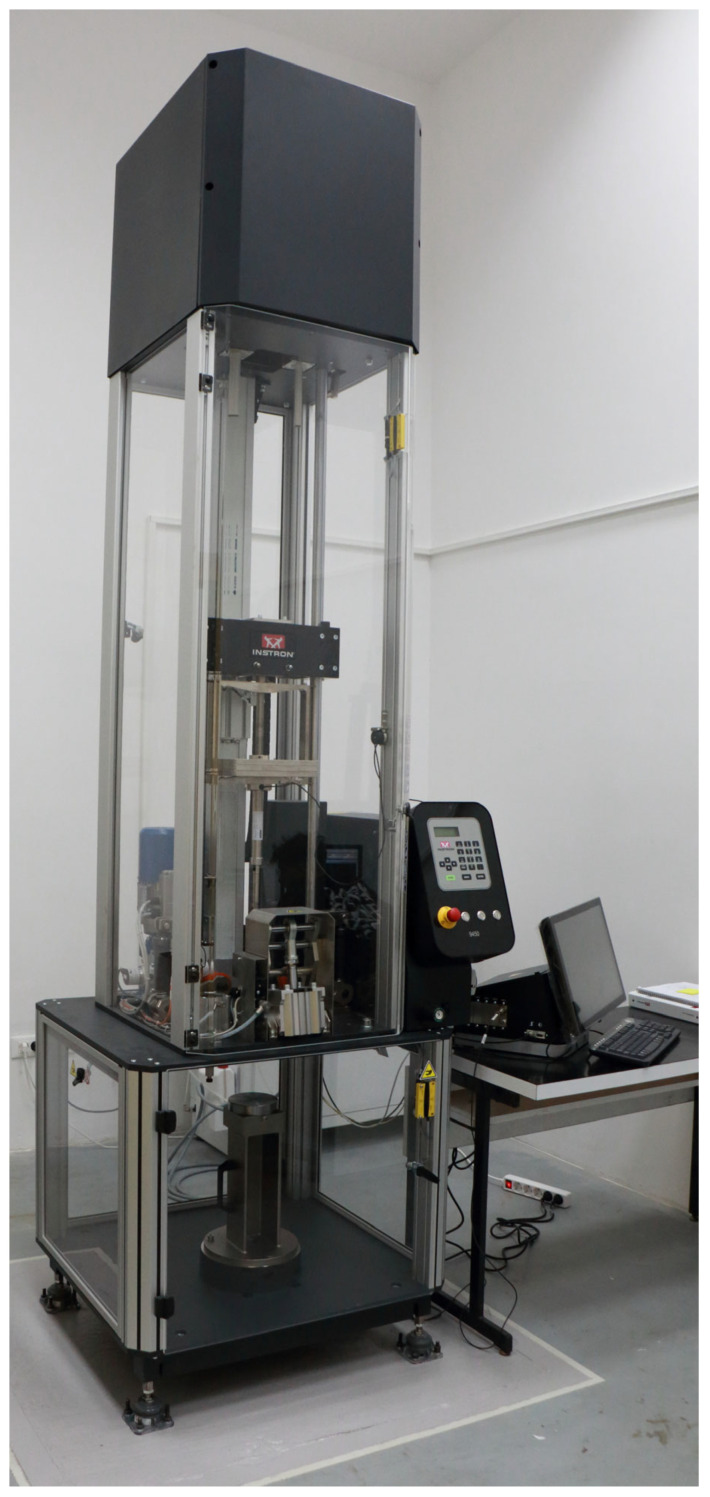
Instron Drop tower 9450 machine.

Two dynamic tests were conducted using the INSTRON Drop Tower 9450 universal testing machine equipped with a 45 kN load cell, Figure 1. The device and load cell are regularly calibrated by the manufacturer to ensure accurate and reliable measurements. The adjustable testing parameters include: impact velocity (ranging from 1 to 24 m/s), drop height (from 0.5 m to 40 m), impactor mass (with additional mass up to 70 kg), impact energy (up to 1800 J), and impactor geometry (wedge-shaped, spherical, or flat-ended). The measured quantity is force, which is recorded by the strain gauge load cell throughout the entire test at an acquisition frequency of up to 4 MHz.

During the execution of dynamic tests, it is necessary to first conduct several preliminary trials, given the lack of available data on this type of testing in the existing literature. Based on the results of the preliminary tests, a detailed testing plan is developed. The plan includes testing multiple femurs at different impact velocity ranges, with four specimens for each set of parameters.

For all dynamic tests, the impact energy was maintained constant to ensure comparability of results, while the impact velocity was used as the variable testing parameter. This approach allows a controlled assessment of the influence of velocity on fracture mechanisms, keeping the total energy input constant. Consequently, the primary variable of interest is the fracture response as a function of impact velocity, and the experimental design ensures that observed differences in fracture behaviour can be attributed predominantly to variations in velocity rather than total energy.

From the obtained force–time data (in tabular .csv format: F, t), the impact energy, impact velocity, absorbed energy, and displacement during impact were calculated using the following expressions:

(1) $$E_{impact}=m_{tot}\times g\times h$$

(2) $$v_{i}=v_{i-1}-t_{sampling}\times \frac{\frac{F_{i}+F_{i-1}}{2}-g\times m_{tot}}{m_{tot}}$$

(3) $$E_{t}=\overset{i-1}{\underset{i=0}{\sum }}E_{i}+t_{sampling}\times \frac{F_{i}v_{i}+F_{i-1}v_{i-1}}{2}$$

(4) $$d_{i}=\overset{i-1}{\underset{i=0}{\sum }}d_{i}+t_{sampling}\times \frac{v_{i}+v_{i-1}}{2}$$

where
$E_{impact}$$\left[J\right]$: impact energy—the total energy available;m$\left[\mathrm{kg}\right]$: total mass of the system;g$\left[m/s^{2}\right]$: gravity acceleration;h$\left[m\right]$: Free-fall heightv$\left[m/s^{2}\right]$: velocity;t$\left[s\right]$: time;F$\left[N\right]$: force;$E_{t}$$\left[J\right]$: absorbed energy;$d_{i}$$\left[\mathrm{mm}\right]$: displacement.

**Figure 2:**
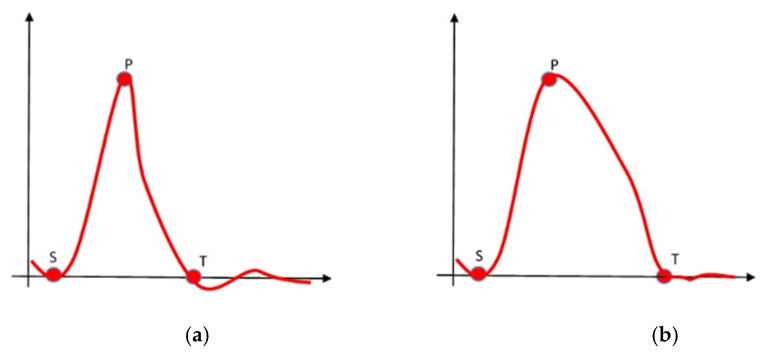
Characteristic force/time diagram for (**a**) brittle fracture, (**b**) ductile fracture.

The ductility index evaluates the propagation behaviour of brittle versus ductile fracture based on direct force measurements from a force/time diagram. The ductility index value is expressed in percentages [%] and is calculated according to the following expression:

(5) $$Ductility\;index\;\left[\%\right]=\;\frac{\left(Energy\;at\;Total\right)-\left(Energy\;at\;Peak\right)}{Energy\;at\;Peak}$$

where the characteristic points in time (Total T and Peak P) are shown in Figure 2.

Ductility is a characteristic of a material that allows it to withstand plastic deformation without breaking. The greater the deformation a material can withstand without brittle fracture, the greater its ductility. If the strain rate is high, the total strain hardening capacity will be quickly exhausted, leading to low ductility of the material. Furthermore, if the yield strength is very high, the strain hardening ability can be reduced, which also leads to lower ductility at the same strain hardening rate. Thus, a lower ductility index suggests that the fracture is closer to a brittle fracture, while a higher ductility index suggests that the fracture is closer to a ductile fracture.

**Figure 3:**
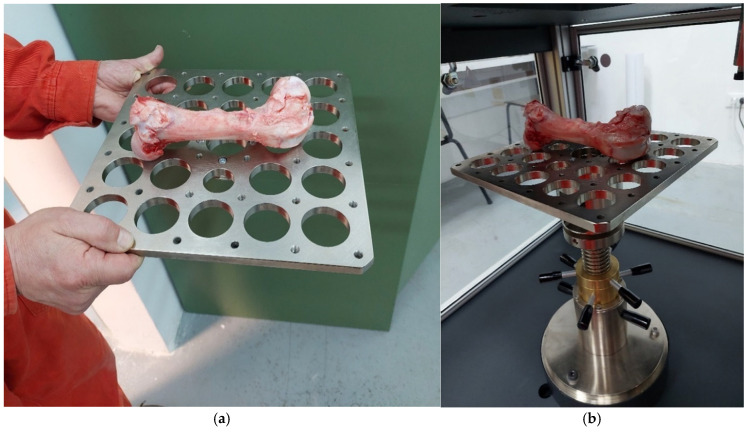
Bone support on a universal table during dynamic testing: (**a**) fixation to universal plate, (**b**) universal stand.

For dynamic tests, a 45 kN impactor, a light frame, a wedge impactor that allows concentrated force along a single impact line, and a universal clamping machine for samples of irregular geometry were used. The bones were clamped on a universal table with two screws attached to the ends of the bones, as shown in Figure 3.

Two sets of dynamic tests were performed where the impact energy E = 70 J was kept as a constant value. In the first set, the impact velocity of the striker was set to 5.15 m/s, and in the second set of dynamic tests, the impact velocity was set to 7.84 m/s. In each set, 4 femurs were tested, resulting in a total of eight femurs tested. In order to maintain the same impact energy, when testing at a lower velocity, a set of weights (+3 kg) was added to the impactor. The test parameters are shown in Table 1.

The use of only eight specimens is justified by the difficulty of obtaining uniform porcine femurs. From a total of 20 fresh femurs, only healthy hind-leg femurs of approximately equal size were selected. Since bone toughness depends strongly on the cross-sectional dimensions at the fracture site, using bones of similar size allows for meaningful comparisons.

## 3. Results

### 3.1. Experiment 1

In the first set of dynamic tests, the impact velocity of the batsman was set at 5.15 m/s. Four femurs were examined in order to characterize the fracture. The descriptive parameter is the ductility index, expressed in percentages, where a higher ductility index indicates that the nature of the fracture is ductile; that is, a lower ductility index indicates that the nature of the resulting fracture is brittle.

In the performed dynamic tests, the impact energy E = 70 J was kept as a constant value. In order to maintain the same impact energy, when testing at a lower velocity, a set of weights (+3 kg) was added to the impactor. The test parameters are given in Table 2.

**Figure 4:**
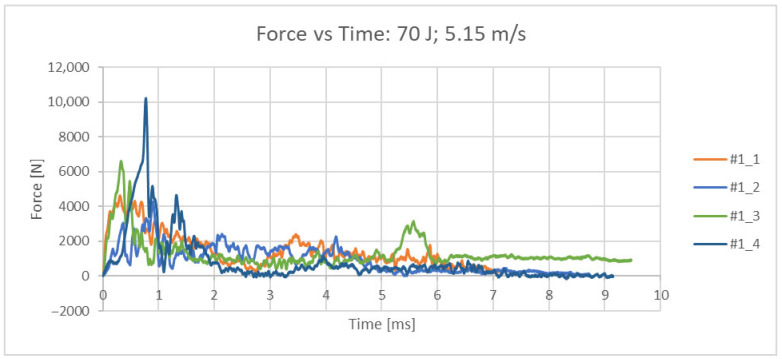
Force/time diagram for all 4 samples at an impact velocity of 5.15 m/s.

**Figure 5:**
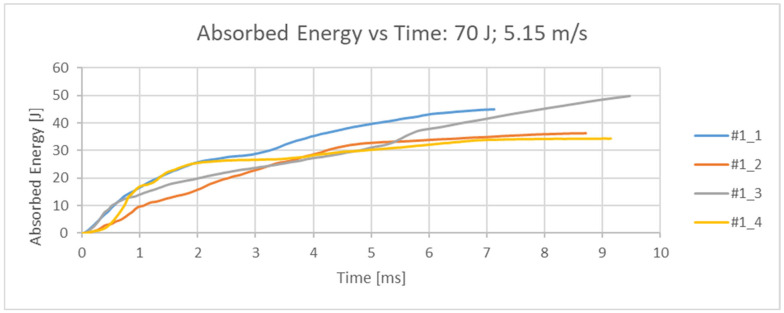
Absorbed energy time diagram for all 4 samples at an impact velocity of 5.15 m/s.

**Figure 6:**
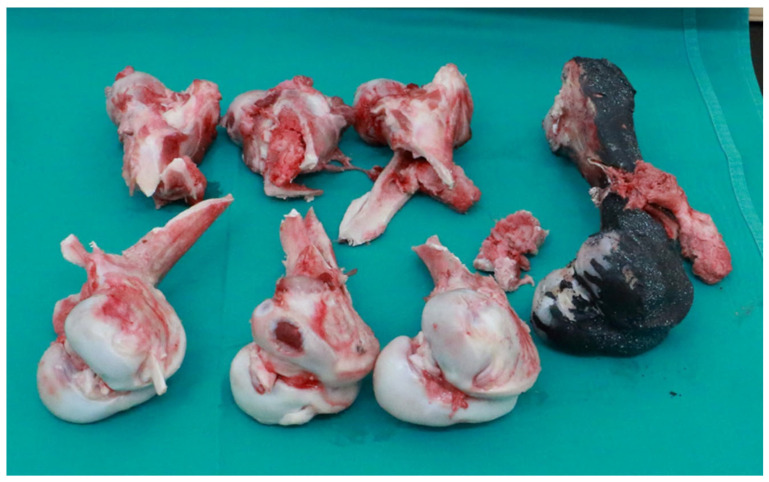
Photos of FEMUR bone after impact test, velocity = 5.15 m/s.

The measured quantity is the force [N], which is directly measured by the strain gauge head of the impactor with an acquisition frequency of 4 MHz. The original set of measurement data (readings from the test results) for all 4 samples is given in the force/time diagram in Figure 4. From the measured values, the absorbed energy over time was calculated, Figure 5. From the measured force, other values of interest were also calculated (maximum impact force, force at the moment of impact, displacement at maximum force, displacement at the moment of failure, energy at the moment of failure, total absorbed energy, ductility index and total impact duration) and are shown in Table 2. The femurus after experiment 1 are shown in Figure 6.

### 3.2. Experiment 2

**Figure 7:**
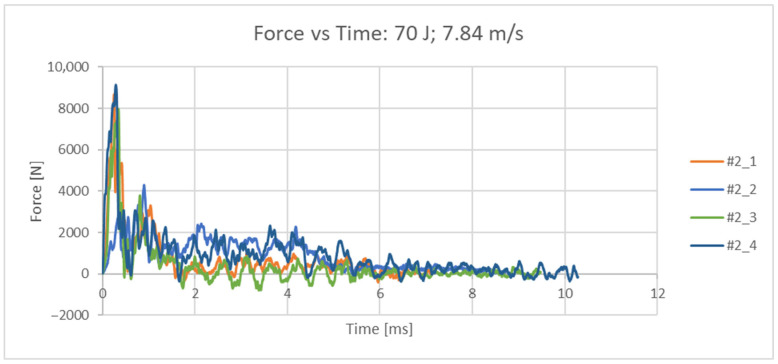
Force/time diagram for all 4 samples at an impact velocity of 7.84 m/s.

**Figure 8:**
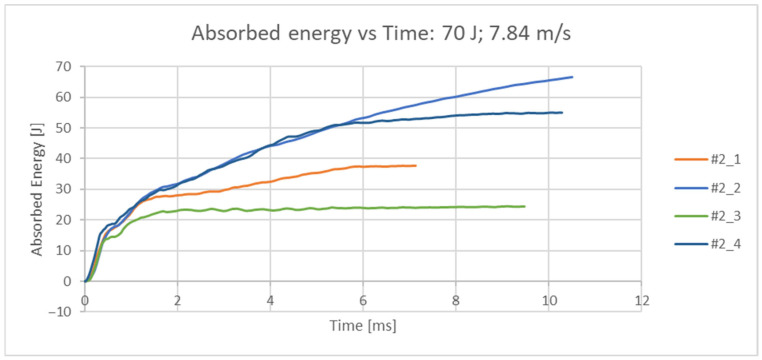
Absorbed energy/time diagram for all 4 samples at an impact velocity of 7.84 m/s.

**Figure 9:**
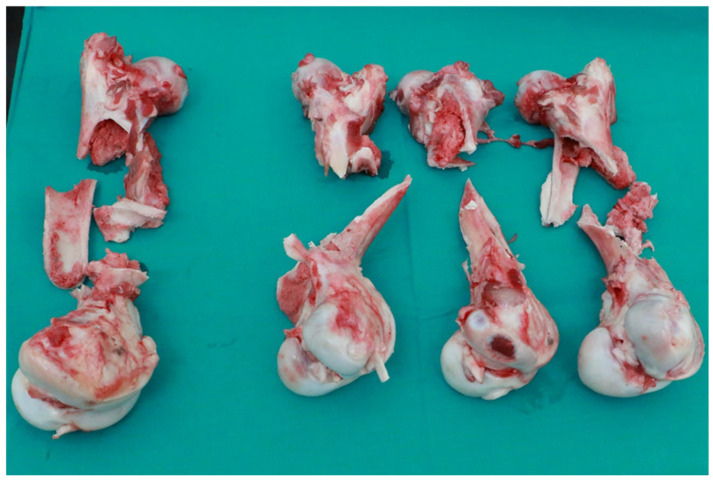
Photos of FEMUR bone after impact test, velocity of 10 m/s.

In the second set of dynamic tests, the impact velocity was set at 7.84 m/s. Four femurs were examined in order to characterize the fracture. The descriptive parameter is the ductility index, expressed in percentages, where a higher ductility index indicates that the nature of the fracture is ductile; that is, a lower ductility index indicates that the nature of the resulting fracture is brittle. In the conducted dynamic tests, the impact energy E = 70 J was kept as a constant value. The measured quantity is the force [N], which is directly measured by the tensometer of the striker head with an acquisition frequency of 4 MHz. The original set of measurement data (readings from the test results) for all 4 samples is given in the force/time diagram in Figure 7. From the measured values, the absorbed energy over time was calculated, Figure 8. From the measured force, other values of interest were also calculated (maximum impact force, force at the moment of impact, displacement at maximum force, displacement at the moment of failure, energy at the moment of failure, total absorbed energy, ductility index and total impact duration) and are shown in Table 3. The femurus after experiment 2 are shown in Figure 9.

The results obtained from the dynamic impact tests indicate noticeable variability in the ductility index among individual femoral specimens. In Experiment 1 (impact velocity 5.15 m/s), bone masses ranged from 268 g to 357 g, while the corresponding ductility indices varied widely between 26.6% and 67.9%, Table 2. In Experiment 2 (impact velocity 7.84 m/s), bone masses were between 289 g and 351 g, and ductility indices ranged from 19.5% to 31.7%, Table 3. Simple Pearson correlation analyses revealed weak and statistically insignificant relationships between bone mass and ductility index within both velocity groups (r ≈ −0.42 at 5.15 m/s and r ≈ +0.16 at 7.84 m/s). Linear regression slopes were −0.189%/g and +0.033%/g, respectively.

## 4. Discussion

Bone is a complex tissue composed of inorganic, organic, and cellular components. It is not an inert structure; rather, it is metabolically active and dynamically remodels throughout life via continuous processes of resorption and renewal.

The term bone quality encompasses multiple aspects that affect a bone’s ability to resist fracture [1,2]. These aspects include structural properties, such as geometry and microarchitecture, as well as material properties, including mineral and collagen composition, across multiple hierarchical levels of organization [2,3]. Bone strength is directly related to its material composition and structural integrity, requiring a balance between stiffness, which provides resistance to deformation, and flexibility, which allows energy absorption through deformation. Following a fracture, the force–displacement curve may exhibit additional displacement due to the release of stored energy or secondary microfractures.

From the obtained force/time data (Table 1, Table 2 and Table 3), impact energy, impact velocity, energy absorbed during impact, displacement, and fracture ductility index were calculated, and corresponding diagrams were generated. The working hypothesis is that different fracture mechanisms occur at different impact velocities. At lower velocities, bone is expected to fracture in a more ductile manner because there is sufficient time for elastic deformation, whereas higher velocities typically result in brittle or even comminuted fractures.

Fracture mechanisms are quantified using the ductility index [%], which reflects a material’s capacity to undergo plastic deformation prior to failure. Greater deformation without brittle fracture corresponds to a higher ductility index. At high strain rates, strain hardening is rapidly exhausted, reducing ductility. Similarly, a high yield strength may limit strain hardening capacity, further decreasing ductility. Consequently, a lower ductility index indicates a fracture closer to brittle behaviour, whereas a higher ductility index corresponds to a tougher fracture.

Cortical bone exhibits viscoelastic-viscoplastic behaviour, with mechanical properties strongly dependent on loading rate. Previous studies have shown that ultimate strength, elastic modulus, and energy required for failure are all rate dependent. Prediction of cancellous bone behaviour is more challenging due to its anisotropic and viscoelastic nature.

From a practical clinical perspective, lower bone mass—reflected by a lower ductility index—is observable on X-ray through cortical thickness and the density of cancellous bone trabeculae, despite the overall optimized bone geometry. There is a direct relationship between the total bone mass (or any energy-absorbing material as a percentage of its volume) and its capacity to absorb energy, analogous to a cushion or spring. Therefore, the ductility index (%) and osteoporosis expressed as a percentage can serve as direct indicators for clinical and practical applications.

## 5. Conclusions

Although these correlations are not robust due to the small sample size (*n* = 4 per group), the substantial variation in ductility among specimens—particularly at the lower impact velocity—suggests that additional intrinsic factors, such as mineral composition, collagen quality, and microstructural organization, play a decisive role in the fracture mechanism rather than bone mass alone.

The variation observed in the ductility index among the tested femurs likely reflects differences not only in bone geometry and density but also in mineral composition and the quality of the organic matrix. Bone mechanical behaviour depends strongly on the mineral-to-collagen ratio and the chemical characteristics of the mineral phase. A reduction in calcium or an altered Ca/P molar ratio is typically associated with lower stiffness and strength, as well as increased solubility of the apatite phase, which can lead to greater brittleness and reduced energy absorption capacity during impact loading [4,5,6].

Furthermore, the substitution of carbonate ions within the hydroxyapatite lattice modifies crystallinity and elasticity. Increased carbonate content generally enhances mineral solubility and decreases the elastic modulus, which can either increase or reduce toughness depending on the extent of substitution [7,8]. Bone crystallinity is another key determinant of fracture behaviour—highly crystalline apatite is mechanically stiffer but more brittle, while lower crystallinity can improve crack resistance at the expense of stiffness [7,8,9].

The quality and cross-linking of collagen fibrils play a critical role in post-yield deformation and energy absorption. Age-related accumulation of advanced glycation end-products (AGEs) and alterations in enzymatic cross-links diminish collagen ductility, leading to brittle fractures even in bones with normal mineral density [10,11,12]. Such degradation of the organic phase could explain part of the observed reduction in the ductility index for specific specimens.

Microarchitectural parameters, such as trabecular number (Tb.N), thickness (Tb.Th), and cortical thickness, are also major determinants of bone mechanical competence. Reduced bone volume fraction (BV/TV) and increased porosity correlate with diminished strength and energy absorption capacity [13,14]. Although these parameters were not directly measured in the present study, their potential influence on ductility should not be disregarded.

Trace elements, particularly magnesium, zinc, and strontium, may further contribute to the observed variability in mechanical response. Magnesium affects crystal size and the degree of mineral lattice substitution, while strontium can enhance bone mineral density and improve mechanical properties by modifying apatite chemistry and stimulating osteoblastic activity [15,16,17].

In summary, the mechanical variability among specimens likely arises from a complex interplay between mineral composition, crystallinity, collagen cross-linking, and microstructural organization. Future studies should therefore include quantitative analyses of Ca/P ratio (via ICP-MS or SEM-EDS), carbonate substitution and crystallinity (via FTIR or XRD), and trabecular/cortical morphology (via micro-CT) to establish stronger correlations between mineral characteristics and ductility indices.

## Figures and Tables

**Table 1:** Test parameters.

Specimen ID	Bone Mass	Velocity	Height	Tup Mass	Additional Mass	Total Mass of System	Energy
	g	m/s	m	kg	kg	kg	J
#1_1	268	5.15	1352.7	2.277	3	5.277	70
#1_2	308	5.15	1352.7	2.277	3	5.277	70
#1_3	357	5.15	1352.7	2.277	3	5.277	70
#1_4	355	5.15	1352.7	2.277	3	5.277	70
#2_1	320	7.84	3134.8	2.277	0	2.277	70
#2_2	301	7.84	3134.8	2.277	0	2.277	70
#2_3	320	7.84	3134.8	2.277	0	2.277	70
#2_4	351	7.84	3134.8	2.277	0	2.277	70

**Table 2:** Values (parameters) recorded during experiment 1.

		Impact Test: E = 70 J, v = 5.15 m/s			
		#1_1	#1_2	#1_3	#1_4
Bone mass	g	268	308	357	355
Impact velocity	m/s	5.15	5.15	5.15	5.15
Impact height	mm	1352.70	1352.70	1352.70	1352.70
Tup mass	kg	2.28	2.28	2.28	2.28
Additional mass	kg	3.00	3.00	3.00	3.00
Total system mass	kg	5.28	5.28	5.28	5.28
Impact energy	J	70.00	70.00	70.00	70.00
Force at Peak	N	5232.219	4298.68	6616.92	10,235.85
Force at Puncture	N	2614.665	2149.17	3305.98	5093.19
Force at Total	N	522.933	421.47	659.53	1002.18
Energy at Peak	J	8.58	8.30	5.77	11.74
Energy at Puncture	J	13.627	9.51	7.77	13.41
Energy at Total	J	26.729	11.30	12.94	17.40
Local energy absorbed S-T	J	26.73	11.30	12.94	17.40
Velocity at Start	m/s	4.89	5.21	5.21	5.21
Velocity at Peak	m/s	4.7	4.91	5.00	4.77
Velocity at Puncture	m/s	4.15	4.86	4.92	4.71
Velocity at Total	m/s	1.06	4.79	4.72	4.54
Velocity variation	m/s	2.444	0.42	0.49	0.67
Displacement at Peak	mm	3.714	4.58	1.69	3.93
Displacement at Puncture	mm	10.334	4.95	2.05	4.13
Displacement at Total	mm	YS	6.26	4.06	5.28
Break type (ISO 6603-2)		ND	YS	YS	YS
Ductility index percentage	%	67.9	26.6	55.4	32.5
Ductility index comment		Ductile	Brittle	Brittle/Ductile	Brittle/Ductile
Slow down	%	20.4	8.00	9.30	12.80
Deceleration—Peak	m/s^2^	991.514	814.61	1253.92	1939.71
Deceleration—Puncture	m/s^2^	495.483	407.27	626.49	965.17

**Table 3:** Values (parameters) recorded during experiment 2.

		Impact Test: E = 70 J, v = 7.84 m/s			
		#2_1	#2_2	#2_3	#2_4
Bone mass	g	289.00	301.00	320.00	351.00
Impact velocity	m/s	7.84	7.84	7.84	7.84
Impact height	mm	3134.80	3134.80	3134.80	3134.80
Tup mass	kg	2.28	2.28	2.28	2.28
Additional mass	kg	0.00	0.00	0.00	0.00
Total system mass	kg	2.28	2.28	2.28	2.28
Impact energy	J	70.00	70.00	70.00	70.00
Force at Peak	N	6077.81	7753.74	7966.49	9139.02
Force at Puncture	N	3015.33	3876.52	3973.71	4556.86
Force at Total	N	594.24	762.04	772.55	910.77
Energy at Peak	J	10.27	12.23	11.08	12.89
Energy at Puncture	J	11.59	13.42	12.21	15.34
Energy at Total	J	13.52	17.92	13.76	18.33
Local energy absorbed S-T	J	13.52	17.92	13.76	18.33
Velocity at Start	m/s	5.21	8.07	8.04	8.07
Velocity at Peak	m/s	4.83	7.38	7.41	7.34
Velocity at Puncture	m/s	4.78	7.31	7.35	7.19
Velocity at Total	m/s	4.70	7.03	7.25	7.01
Velocity variation	m/s	0.51	1.04	0.79	1.06
Displacement at Peak	mm	2.91	2.96	2.73	2.24
Displacement at Puncture	mm	3.18	3.15	2.90	2.56
Displacement at Total	mm	4.12	4.94	3.54	3.83
Break type (ISO 6603-2)		YS	YS	YS	YS
Ductility index percentage	%	24	31.70	19.50	29.70
Ductility index comment		Brittle	Brittle/Ductile	Brittle	Brittle
Slow down	%	9.80	12.80	9.80	13.20
Deceleration—Peak	m/s^2^	1151.76	3405.24	3498.68	4013.62
Deceleration—Puncture	m/s^2^	571.41	1702.47	1745.15	2001.25

## Data Availability

Data will be available upon request.
